# Supplementary material for: Where is emotional feeling felt in the body? An integrative review
Source: PLoS One. 2021 Dec 22;16(12):e0261685. doi: 10.1371/journal.pone.0261685 (PMC8694467; doi:10.1371/journal.pone.0261685)
Supplement: S1 Table — (DOCX) [file pone.0261685.s001.docx]

**Search strategies (4 stages)**

Each ‘level’ indicates the separate searches undertaken within each database’s search function to arrive at the final number of hits. The notation used in each table pertains to the idiosyncrasies of each database.

**First stage:** *large scale library-led search strategy*

*Medline, PsycInfo, EMBASE*

| Level | Block of search terms |
| --- | --- |
| 1 | ((feeling* or emotion* or affect) adj1 (embod* or ((body or bodily or physiolog*) adj2 (manifest* or localis* or process* or structur* or boundar* or location* or located or present* or condition or change* or experienc* or express* or root*)))).ti. or ((feeling* or emotion* or affect) adj1 (embod* or ((body or bodily or physiolog*) adj2 (manifest* or localis* or process* or structur* or boundar* or location* or located or present* or condition or change* or experienc* or express* or root*)))).ab. /freq=2 |
| 2 | "core affect*".tw. |
| 3 | (interocept* or physiologic* or visceral or viscera or embod* or body or bodily or somatic* or heart or hearts or cardio* or cardiac* or gut or gastro* or intestin* or "smooth muscle*" or lung* or respiratory or respiration or organ or organs or stomach or "vagus nerve" or "enteric nervous").tw. |
| 4 | 2 and 3 |
| 5 | "embodied emotion*".mp. |
| 6 | "William James".ab. and (emotion* or affect* or feeling* or embod*).tw. |
| 7 | 1 or 4 or 5 or 6 |
| 8 | limit 7 to English language |

*EBSCO (Psychiatry and Behavioural Sciences Collection)*

| Level | Block of search terms |
| --- | --- |
| S1 | TI ((feeling* or emotion* or affect) N1 (embod* or body or bodily or physiolog*) N2 (manifest* or localis* or process* or structur* or boundar* or location* or located or present* or condition or change* or experienc* or express* or root*)) OR AB ((feeling* or emotion* or affect) N1 (embod* or body or bodily or physiolog*) N2 (manifest* or localis* or process* or structur* or boundar* or location* or located or present* or condition or change* or experienc* or express* or root*)) OR SU ((feeling* or emotion* or affect) N1 (embod* or body or bodily or physiolog*) N2 (manifest* or localis* or process* or structur* or boundar* or location* or located or present* or condition or change* or experienc* or express* or root*)) |
| S2 | TX "core affect*" |
| S3 | TI (interocept* or physiologic* or visceral or viscera or embod* or body or bodily or somatic* or heart or hearts or cardio* or cardiac* or gut or gastro* or intestin* or "smooth muscle*" or lung* or respiratory or respiration or organ or organs or stomach or "vagus nerve" or "enteric nervous") OR AB (interocept* or physiologic* or visceral or viscera or embod* or body or bodily or somatic* or heart or hearts or cardio* or cardiac* or gut or gastro* or intestin* or "smooth muscle*" or lung* or respiratory or respiration or organ or organs or stomach or "vagus nerve" or "enteric nervous") OR SU (interocept* or physiologic* or visceral or viscera or embod* or body or bodily or somatic* or heart or hearts or cardio* or cardiac* or gut or gastro* or intestin* or "smooth muscle*" or lung* or respiratory or respiration or organ or organs or stomach or "vagus nerve" or "enteric nervous") |
| S4 | S2 AND S3 |
| S5 | AB "William James" |
| S6 | TI (emotion* or affect* or feeling* or embod*) OR AB (emotion* or affect* or feeling* or embod*) OR SU (emotion* or affect* or feeling* or embod*) |
| S7 | S5 AND S6 |
| S8 | S1 OR S4 OR S7 |

*SCOPUS*

| Level | Block of search terms |
| --- | --- |
| 1 | (TITLE (embodied AND (mind OR emotion OR cognition)) OR TITLE (interocept*) OR TITLE ("aesthetic experience")) |
| 2 | (locali* OR locus OR locat* OR stomach OR gut OR body) |
| 3 | NOT ((TITLE (brain* OR cerebellum)) OR (TITLE-ABS-KEY (computer* OR robot* OR haptic*) OR SRCTITLE (comput* OR robot* OR haptic* OR brain))) |
| 4 | EXCLUDE (DOCTYPE, “cp”) OR EXCLUDE (DOCTYPE, “ed”) OR EXCLUDE (DOCTYPE, “le”) |
| Other limits | English language; peer reviewed; psychology, neuroscience or medicine |

*Cochrane database of systematic reviews; Cochrane Central Register of Controlled Trials*

| Level | Block of search terms |
| --- | --- |
| 1 | Embodied |
| 2 | (mind OR emotion OR cognition) OR (interocept*) |
| 3 | local* OR locat* OR stomach OR gut OR body |

**Second stage:** *Gut-focussed searches*

*All databases*

| Level | Block of search terms |
| --- | --- |
| 1 | Emotion* OR “core affect” OR affective OR anxiety OR threat OR panic OR stress |
| 2 | Interocept* OR mindfulness OR “somatic symptom” OR embod* |
| 3 | Feeling OR *sensitivity OR sensitiv* OR sensation OR arousal |
| 4 | Gut OR gastro* OR gastric OR bowel OR stomach OR intestin* OR colon OR enteric |

**Third stage**: *reference lists*

**Fourth stage:** *Core text measurement tool searches*

*Body mapping (all databases)*

| Measurement tool | Block of search terms |
| --- | --- |
| 1 | Emotion* OR “core affect” OR affective |
| 2 | Feeling OR sensitivity OR sensitiv* OR sensation OR arousal |
| 3 | “Body map*” OR “emBODY” OR “topographical map” |

*Scene Construction Questionnaire (all databases)*

| Measurement tool | Block of search terms |
| --- | --- |
| 1 | Emotion* OR “core affect” OR affective OR anxiety OR threat OR panic OR stress |
| 2 | Feeling OR sensitivity OR sensitiv* OR sensation OR arousal OR symptom* |
| 3 | “Scene Construction Questionnaire” OR “SCQ” |

*Visual Analogue Scale with self-report checklist (all databases)*

| Measurement tool | Block of search terms |
| --- | --- |
| 1 | Emotion* OR “core affect” OR affective OR anxiety OR threat OR panic OR stress) |
| 2 | “VAS” OR “Visual Analogue Scale*” |
| 3 | (Feeling OR sensitivity OR sensitiv* OR sensation OR arousal OR symptom*) AND (“Hubert” OR “de Jong-Meyer”) |
