# Supplementary material for: Where is emotional feeling felt in the body? An integrative review
Source: PLoS One. 2021 Dec 22;16(12):e0261685. doi: 10.1371/journal.pone.0261685 (PMC8694467; doi:10.1371/journal.pone.0261685)
Supplement: S2 Table — (DOCX) [file pone.0261685.s002.docx]

**Core text location summaries**

*Body map study locations indicated activated (A) or deactivated (D) or neutral (N); (T) denotes possible “traces” of activation: Anger*

| Publication | *Head/*  *face* | *Chest* | *Throat* | *Upper arms* | *Lower arms* | *Hands* | Lower abdomen | *Groin* | Upper legs | *Lower legs* | Feet |
| --- | --- | --- | --- | --- | --- | --- | --- | --- | --- | --- | --- |
| Torregrossa, L. J., Snodgress, M. A., Hong, S. J., Nichols, H. S., Glerean, E., Nummenmaa, L., & Park, S. (2019) [text’s Fig. 2., controls] | ✓(A) | ✓(A) | (T) | ✓(A) | ✓(A) | ✓(A) | (N) | (N) | (T) | (N) | (N) |
| Nummenmaa, L., Glerean, E., Hari, R., & Hietanen, J. K. (2014) [text’s Fig 2., word stimuli] | ✓(A) | ✓(A) | ✓(A) | ✓(A) | ✓(A) | ✓(A) | (T) | (N) | (T) | (T) | ✓(A) |
| Hietanen, Glerean, Hari & Nummenmaa, (2016) [text’s Fig 2., adults] | ✓(A) | ✓(A) | ✓(A) | ✓(A) | ✓(A) | ✓(A) | (T) | (N) | (N) | (T) | ✓(A) |
| Sachs, M. E., Kaplan, J., & Habibi, A. (2019) [text’ Fig. 1., self, adults; music or film] | ✓(A) | ✓(A) | ✓(A) | ✓(A) | (N) | (T) | (N) | (N) | (N) | (N) | (N) |
| Novembre, G., Zanon, M., Morrison, I., & Ambron, E. (2019) [text’s Fig. 4] | ✓(A) | ✓(A) | ✓(A) | ✓(A) | ✓(A) | ✓(A) | (T) | (N) | (T) | (N) | (T) |
| Volynets, S., Glerean, E., Hietanen, J. K., Hari, R., & Nummenmaa, L. (2019) [text’s Fig 2.] | ✓(A) | ✓(A) | ✓(A) | ✓(A) | ✓(A) | ✓(A) | ✓(A) | (N) | (T) | (T) | (T) |
| Jung, W. M., Ryu, Y., Lee, Y. S., Wallraven, C., & Chae, Y. (2017) [text’s Fig. 2 (Z-scores); “sensation” only: 4 = high z-score] | ✓(4) | ✓(4) | ✓ | ✓ | (T) | ✓ | ✓ | ✓ | ✓ | (N) | (T) |

*Body map study locations indicated activated (A) or deactivated (D) or neutral (N); (T) denotes possible “traces” of activation: Fear*

| Publication | *Head/*  *face* | *Chest* | *Throat* | Upper arms | *Lower arms* | Hands | *Lower abdomen* | *Groin* | *Upper legs* | Lower legs | Feet |
| --- | --- | --- | --- | --- | --- | --- | --- | --- | --- | --- | --- |
| Torregrossa, L. J., Snodgress, M. A., Hong, S. J., Nichols, H. S., Glerean, E., Nummenmaa, L., & Park, S. (2019) [text’s Fig. 2., controls] | ✓(A) | ✓(A) | (N) | ✓(A) | (N) | (N) | ✓(A) | (N) | (N) | (N) | (N) |
| Nummenmaa, L., Glerean, E., Hari, R., & Hietanen, J. K. (2014) [text’s Fig 2., word stimuli] | ✓(A) | ✓(A) | ✓(A) | (T) | (T) | ✓(A) | ✓(A) | (N) | (N) | (T) | (T) |
| Hietanen, Glerean, Hari & Nummenmaa, (2016) [text’s Fig 2., adults] | ✓(A) | ✓(A) | ✓(A) | ✓(A) | (T) | (N) | ✓(A) | (T) | ✓(A) | ✓(A) | ✓(A) |
| Sachs, M. E., Kaplan, J., & Habibi, A. (2019) [text’ Fig. 1., self, adults; music or film] | ✓(A) | ✓(A) | ✓(A) | (N) | (N) | (N) | ✓(A) | (N) | (N) | (N) | ✓(A) |
| Novembre, G., Zanon, M., Morrison, I., & Ambron, E. (2019) [text’s Fig. 4] | (T) | ✓(A) | (T) | (N) | (N) | (N) | (T) | (N) | (N) | (N) | (N) |
| Volynets, S., Glerean, E., Hietanen, J. K., Hari, R., & Nummenmaa, L. (2019) [text’s Fig 2.] | ✓(A) | ✓(A) | ✓(A) | (T) | (N) | (T) | ✓(A) | (N) | (N) | (N) | (N) |
| Jung, W. M., Ryu, Y., Lee, Y. S., Wallraven, C., & Chae, Y. (2017) [text’s Fig. 2 (Z-scores); “sensation” only: 4 = high z-score] | ✓(4) | ✓(4) | ✓ | ✓ | (T) | ✓ | ✓ | (N) | (T) | ✓ | ✓ |

*Body map study locations indicated activated (A) or deactivated (D) or neutral (N); (T) denotes possible “traces” of activation: Sadness*

| Publication | *Head/*  *face* | Chest | Throat | Upper arms | *Lower arms* | *Hands* | Lower abdomen | Groin | *Upper legs* | *Lower legs* | Feet |
| --- | --- | --- | --- | --- | --- | --- | --- | --- | --- | --- | --- |
| Torregrossa, L. J., Snodgress, M. A., Hong, S. J., Nichols, H. S., Glerean, E., Nummenmaa, L., & Park, S. (2019) [text’s Fig. 2., controls] | (T) | ✓(D) | ✓(D) | (N) | ✓(D) | ✓(D) | ✓(D) | (N) | ✓(D) | ✓(D) | ✓(D) |
| Nummenmaa, L., Glerean, E., Hari, R., & Hietanen, J. K. (2014) [text’s Fig 2., word stimuli] | ✓(A) | ✓(A) | ✓(A) | ✓(D) | ✓(D) | ✓(D) | (T) | ✓(D) | ✓(D) | ✓(D) | ✓(D) |
| Hietanen, Glerean, Hari & Nummenmaa, (2016) [text’s Fig 2., adults] | (T) | ✓(A) | ✓(A) | ✓(D) | ✓(D) | ✓(D) | ✓(A) | ✓(D) | ✓(D) | ✓(D) | ✓(D) |
| Sachs, M. E., Kaplan, J., & Habibi, A. (2019) [text’ Fig. 1., self, adults; music or film] | ✓(A) | ✓(A) | ✓(A) | (N) | (N) | (N) | (T) | (N) | (T) | (T) | (N) |
| Novembre, G., Zanon, M., Morrison, I., & Ambron, E. (2019) [text’s Fig. 4] | (T) | (N) | (N) | ✓(D) | ✓(D) | (T) | (T) | ✓(D) | ✓(D) | ✓(D) | ✓(D) |
| Volynets, S., Glerean, E., Hietanen, J. K., Hari, R., & Nummenmaa, L. (2019) [text’s Fig 2.] | (N) | ✓(A) | (N) | ✓(D) | ✓(D) | ✓(D) | (N) | ✓(D) | ✓(D) | ✓(D) | ✓(D) |
| Jung, W. M., Ryu, Y., Lee, Y. S., Wallraven, C., & Chae, Y. (2017) [text’s Fig. 2 (Z-scores); “sensation” only: 4 = high z-score] | ✓(4) | ✓(4) | (N) | ✓ | (T) | ✓ | ✓ | (N) | (N) | (N) | (N) |

*Body map study locations indicated activated (A) or deactivated (D) or neutral (N); (T) denotes possible “traces” of activation: Disgust*

| Publication | *Head/*  *face* | Chest | *Throat* | *Upper arms* | *Lower arms* | *Hands* | *Lower abdomen* | *Groin* | *Upper legs* | Lower legs | *Feet* |
| --- | --- | --- | --- | --- | --- | --- | --- | --- | --- | --- | --- |
| Torregrossa, L. J., Snodgress, M. A., Hong, S. J., Nichols, H. S., Glerean, E., Nummenmaa, L., & Park, S. (2019) [text’s Fig. 2., controls] | ✓(A) | (N) | (N) | (N) | (N) | (N) | ✓(D) | (N) | (N) | (N) | (N) |
| Nummenmaa, L., Glerean, E., Hari, R., & Hietanen, J. K. (2014) [text’s Fig 2., word stimuli] | ✓(A) | ✓(A) | ✓(A) | (T) | (T) | ✓(A) | ✓(A) | (T) | (N) | (N) | (N) |
| Hietanen, Glerean, Hari & Nummenmaa, (2016) [text’s Fig 2., adults] | ✓(A) | ✓(A) | ✓(A) | (T) | (T) | (T) | ✓(A) | (N) | (N) | (T) | (T) |
| Sachs, M. E., Kaplan, J., & Habibi, A. (2019) [text’ Fig. 1., self, adults; music or film] | N/A | N/A | N/A | N/A | N/A | N/A | N/A | N/A | N/A | N/A | N/A |
| Novembre, G., Zanon, M., Morrison, I., & Ambron, E. (2019) [text’s Fig. 4] | ✓(A) | (N) | ✓(A) | (N) | (N) | (N) | (T) | (N) | (T) | (T) | (N) |
| Volynets, S., Glerean, E., Hietanen, J. K., Hari, R., & Nummenmaa, L. (2019) [text’s Fig 2.] | ✓(A) | ✓(A) | ✓(A) | (N) | (N) | (N) | ✓(A) | (T) | ✓(D) | ✓(D) | (T) |
| Jung, W. M., Ryu, Y., Lee, Y. S., Wallraven, C., & Chae, Y. (2017) [text’s Fig. 2 (Z-scores); “sensation” only: 4 = high z-score] | ✓(4) | ✓(4) | ✓(4) | ✓ | (T) | (N) | ✓ | (N) | (N) | (N) | (N) |

*Body map study locations indicated activated (A) or deactivated (D) or neutral (N); (T) denotes possible “traces” of activation: Happiness*

| Publication | *Head/*  *face* | *Chest* | *Throat* | *Upper arm*s | Lower arms | *Hands* | *Lower abdomen* | Groin | Upper legs | Lower legs | Feet |
| --- | --- | --- | --- | --- | --- | --- | --- | --- | --- | --- | --- |
| Torregrossa, L. J., Snodgress, M. A., Hong, S. J., Nichols, H. S., Glerean, E., Nummenmaa, L., & Park, S. (2019) [text’s Fig. 2., controls] | ✓(A) | ✓(A) | ✓(A) | (T) | (N) | ✓(A) | ✓(A) | ✓(A) | (N) | (N) | (N) |
| Nummenmaa, L., Glerean, E., Hari, R., & Hietanen, J. K. (2014) [text’s Fig 2., word stimuli] | ✓(A) | ✓(A) | ✓(A) | ✓(A) | ✓(A) | ✓(A) | ✓(A) | ✓(A) | ✓(A) | ✓(A) | ✓(A) |
| Hietanen, Glerean, Hari & Nummenmaa, (2016) [text’s Fig 2., adults] | ✓(A) | ✓(A) | ✓(A) | ✓(A) | ✓(A) | ✓(A) | ✓(A) | ✓(A) | ✓(A) | ✓(A) | ✓(A) |
| Sachs, M. E., Kaplan, J., & Habibi, A. (2019) [text’ Fig. 1., self, adults; music or film] | ✓(A) | ✓(A) | ✓(A) | ✓(A) | (N) | (T) | (N) | (N) | (N) | (N) | ✓(A) |
| Novembre, G., Zanon, M., Morrison, I., & Ambron, E. (2019) [text’s Fig. 4] | ✓(A) | ✓(A) | ✓(A) | ✓(A) | ✓(A) | (T) | (T) | ✓(A) | ✓(A) | ✓(A) | (T) |
| Volynets, S., Glerean, E., Hietanen, J. K., Hari, R., & Nummenmaa, L. (2019) [text’s Fig 2.] | ✓(A) | ✓(A) | ✓(A) | ✓(A) | ✓(A) | ✓(A) | ✓(A) | ✓(A) | ✓(A) | ✓(A) | ✓(A) |
| Jung, W. M., Ryu, Y., Lee, Y. S., Wallraven, C., & Chae, Y. (2017) [text’s Fig. 2 (Z-scores); “sensation” only: 4 = high z-score] | ✓(4) | ✓(4) | ✓ | ✓ | (T) | (T) | ✓ | (N) | ✓ | (N) | (N) |

*Body map study locations indicated activated (A) or deactivated (D) or neutral (N); (T) denotes possible “traces” of activation: Surprise*

| Publication | *Head/*  *face* | *Chest* | *Throat* | *Upper arms* | *Lower arms* | *Hands* | Lower abdomen | *Groin* | *Upper legs* | *Lower legs* | *Feet* |
| --- | --- | --- | --- | --- | --- | --- | --- | --- | --- | --- | --- |
| Torregrossa, L. J., Snodgress, M. A., Hong, S. J., Nichols, H. S., Glerean, E., Nummenmaa, L., & Park, S. (2019) [text’s Fig. 2., controls] | ✓(A) | ✓(A) | (N) | (N) | (N) | (N) | (N) | (N) | (N) | (N) | (N) |
| Nummenmaa, L., Glerean, E., Hari, R., & Hietanen, J. K. (2014) [text’s Fig 2., word stimuli] | ✓(A) | ✓(A) | ✓(A) | (T) | (N) | (T) | (T) | (N) | ✓(D) | ✓(D) | (N) |
| Hietanen, Glerean, Hari & Nummenmaa, (2016) [text’s Fig 2., adults] | ✓(A) | ✓(A) | ✓(A) | (T) | (T) | (T) | ✓(A) | (N) | (N) | (N) | (N) |
| Sachs, M. E., Kaplan, J., & Habibi, A. (2019) [text’ Fig. 1., self, adults; music or film] | N/A | N/A | N/A | N/A | N/A | N/A | N/A | N/A | N/A | N/A | N/A |
| Novembre, G., Zanon, M., Morrison, I., & Ambron, E. (2019) [text’s Fig. 4] | ✓(A) | ✓(A) | ✓(A) | (T) | (N) | (N) | (T) | (N) | (T) | (T) | (N) |
| Volynets, S., Glerean, E., Hietanen, J. K., Hari, R., & Nummenmaa, L. (2019) [text’s Fig 2.] | ✓(A) | ✓(A) | ✓(A) | ✓(A) | (T) | ✓(A) | ✓(A) | (N) | (N) | (T) | (N) |
| Jung, W. M., Ryu, Y., Lee, Y. S., Wallraven, C., & Chae, Y. (2017) [text’s Fig. 2 (Z-scores); “sensation” only: 4 = high z-score] | N/A | N/A | N/A | N/A | N/A | N/A | N/A | N/A | N/A | N/A | N/A |

*Body map study locations indicated activated (A) or deactivated (D) or neutral (N); (T) denotes possible “traces” of activation: Neutral*

| Publication | *Head/*  *face* | Chest | *Throat* | *Upper arms* | *Lower arms* | *Hands* | *Lower abdomen* | *Groin* | *Upper legs* | *Lower legs* | *Feet* |
| --- | --- | --- | --- | --- | --- | --- | --- | --- | --- | --- | --- |
| Torregrossa, L. J., Snodgress, M. A., Hong, S. J., Nichols, H. S., Glerean, E., Nummenmaa, L., & Park, S. (2019) [text’s Fig. 2., controls] | (N) | (N) | (N) | (N) | (N) | (N) | (N) | (N) | (N) | (N) | (N) |
| Nummenmaa, L., Glerean, E., Hari, R., & Hietanen, J. K. (2014) [text’s Fig 2., word stimuli] | (N) | ✓(D) | (N) | (T) | (T) | (N) | (N) | (N) | (T) | (T) | (N) |
| Hietanen, Glerean, Hari & Nummenmaa, (2016) [text’s Fig 2., adults] | (N) | ✓(D) | (N) | ✓(D) | (N) | (N) | (N) | (N) | ✓(D) | ✓(D) | ✓(D) |
| Sachs, M. E., Kaplan, J., & Habibi, A. (2019) [text’ Fig. 1., self, adults; music or film] | N/A | N/A | N/A | N/A | N/A | N/A | N/A | N/A | N/A | N/A | N/A |
| Novembre, G., Zanon, M., Morrison, I., & Ambron, E. (2019) [text’s Fig. 4] | N/A | N/A | N/A | N/A | N/A | N/A | N/A | N/A | N/A | N/A | N/A |
| Volynets, S., Glerean, E., Hietanen, J. K., Hari, R., & Nummenmaa, L. (2019) [text’s Fig 2.] | (T) | (N) | (N) | (N) | (N) | (N) | (N) | (N) | (N) | (T) | (N) |
| Jung, W. M., Ryu, Y., Lee, Y. S., Wallraven, C., & Chae, Y. (2017) [text’s Fig. 2 (Z-scores); “sensation” only: 4 = high z-score] | ✓(4) | ✓ | ✓ | (T) | (T) | (T) | ✓ | (N) | (N) | (N) | (T) |
