# Supplementary material for: Where is emotional feeling felt in the body? An integrative review
Source: PLoS One. 2021 Dec 22;16(12):e0261685. doi: 10.1371/journal.pone.0261685 (PMC8694467; doi:10.1371/journal.pone.0261685)
Supplement: S3 Table — (DOCX) [file pone.0261685.s003.docx]

*Locations indicated in traditional self-report papers*

| Publication | Head/face | Chest | Throat | Arms | Hands | Lower abdomen |
| --- | --- | --- | --- | --- | --- | --- |
| Hubert, W., & de Jong-Meyer, R. (1990). Psychophysiology response patterns to positive and negative film stimuli^a^ | Self-report: “blood rushing to head” (significant correlation with skin responses). Physio: negative stimuli showed increased EMG (corrugator), but decreases for positive stimuli; EMG (zygomaticus), increased activity for positive stimuli | Self-report: negative stimuli increased “heart rate increasing”, “faster breathing”. Physio: positive and negative stimuli increased respiration; negative stimuli decreased heart rate. Correlations: (anxious and low anxious groups) for disgust and "shallow breathing", between anxiety and “heart rate increases” and breathing (i.e. “difficulties in breathing” for anxiety, “shallow breathing” for low anxiety). Anxious group: joy with "difficulties in breathing" and "shallow breathing"; negative correlation between anxiety and respiratory activity; disgust: negative correlation with respiration | Not recorded | Not recorded | N/A | Self-report: negative stimuli increased “tense stomach”; decreases for positive stimuli. Correlations: (anxious group) anxiety and “tense stomach”; disgust and "tense stomach" (both groups) |
| Hubert, W., & de Jong-Meyer, R. (1991). Autonomic, neuroendocrine, and subjective responses to emotion-inducing film stimuli^b^ | Self-report: suspense stimuli and “hot face” | Self-report: suspense stimuli and “heart rate increasing”; “difficulties in breathing”. Physio: suspense and pleasant stimuli, elicited heart rate decreases | Not recorded | Not recorded | Self-report: suspense stimuli and “sweaty palms” | N/A |
| Bergquist, K. L., Fox, H. C., & Sinha, R. (2010). Self-Reports of Interoceptive Responses During Stress and Drug Cue-Related Experiences… [“stress” stimuli; shows % of participants reporting symptom]^c^ | Tightness in face (55.4%); grit teeth (55.4%); forehead tension (53.6%); tears (50.0%); clenched jaw (44.6%); eyes water (39.3%); head pounds (32.1%); blood rushes to head (28.6%); flushed face (23.2%); eyes burn (16.1%) | Heart (91.1%); respiratory (breathing; 78.6%); chest (35.7%); tension in back (28.6%) | Lump in throat (26.8%) | Tension in arms (17.9%) | Clenched fist (35.7%); hands trembling (30.4%), palms clammy (25.0%), whole | Gut (stomach; 71.4%) |

^a^ Skin conductance level increased during both stimuli. Joy was positively correlated with skin conductance level. Correlations between subjective body locations/sensations and objective measures in the anxious group: skin conductance level and “heart rate increasing” (0.57), “breathing faster” (0.61) and “tense stomach” (0.76); "spontaneous fluctuation" and shallow breathing (0.54), heart rate increasing (0.75), breathing faster (0.81), blood rushing to head (0.61), tense stomach (0.65).

^b^ Other symptoms overall: “sweating” and “restlessness”. Pleasant films: increases in “restlessness”. Physiological changes (film x time interactions): skin conductance and SF. For skin conductance: increases in suspense, decreases during pleasant films.

^c^ Other symptoms reported: skin (“sweating”; 67.9%), “feel tense all over “(67.9%), “feel hot all over” (41.1%), and “body shakes” (21.4%).

N.B. data on the groin, upper legs, lower legs, feet were not captured by these studies, nor was a distinction between upper and lower arms.
